# Research on the Forward Solving Method of Defect Leakage Signal Based on the Non-Uniform Magnetic Charge Model

**DOI:** 10.3390/s23136221

**Published:** 2023-07-07

**Authors:** Pengfei Gao, Hao Geng, Lijian Yang, Yuming Su

**Affiliations:** College of Information Science and Engineering, Shenyang University of Technology, Shenyang 110870, China; hg202105@163.com (P.G.); yanglijian888@163.com (L.Y.); suhei315@163.com (Y.S.)

**Keywords:** magnetic dipole, magnetic monopoles density, leakage magnetic field calculation, rectangular defects, magnetic leakage signal

## Abstract

Pipeline magnetic flux leakage inspection is widely used in the evaluation of material defect detection due to its advantages of having no coupling agent and easy implementation. The quantification of defect size is an important part of magnetic flux leakage testing. Defects of different geometrical dimensions produce signal waveforms with different characteristics after excitation. The key to achieving defect quantification is an accurate description of the relationship between the magnetic leakage signal and the size. In this paper, a calculation model for solving the defect leakage field based on the non-uniform magnetic charge distribution of magnetic dipoles is developed. Based on the traditional uniformly distributed magnetic charge model, the magnetic charge density distribution model is improved. Considering the variation of magnetic charge density with different depth positions, the triaxial signal characteristics of the defect are obtained by vector synthesis calculation. Simultaneous design of excitation pulling experiment. The leakage field distribution of rectangular defects with different geometries is analyzed. The experimental results show that the change in defect size will have an impact on the area of the defect leakage field distribution, and the larger the length and wider the width of the defect, the more sensitive the impact on the leakage field distribution. The solution model is consistent with the experimentally obtained leakage signal distribution law, and the model is a practical guide by which to improve the quality of defect evaluation.

## 1. Introduction

In the field of magnetic flux leakage testing, determining the defect size based on defect leakage signals is an important aspect of the inspection process. To establish a solution model that accurately describes the relationship between the spatial magnetic field and the defect size, it is crucial to establish a solution model that accurately reflects the objective facts, minimizes errors, and effectively describes the relationship between the magnetic signal leakage of the pipe and the size of the defect. This is essential for conducting an analytical study on the characteristics of the defect signal. The leakage signal is influenced by various practical factors, such as the defect size, the sensor detection angle, the external magnetization strength, the material properties of the measured object, and the detection speed. B Nestleroth collected a large number of metal defects of different sizes by means of a pipe detector and investigated the dimensional relationship between the data under the pipe detector and the metal defects [1,2]. Sherbinin studied the relationship between the crack defect depth and the leakage signal, and provided the expected quantification formula for crack characteristics [3,4]. G. Katragadda et al. investigated the difference in the leakage magnetic signals of cracks distributed along the entire pipeline circumference and cracks of finite length using the finite element method [5,6]. Jiang Qi et al. defined the defect profile size for oil and gas pipeline corrosion defects and produced a large number of different categories of sample defects for analysis, providing the relationship curves between the defect profile and leakage magnetic field characteristics, and experimentally concluded that these characteristics could effectively quantify the defect profile size [7]. According to the theory of magnetic dipole, Weichang proved that due to the different shapes, magnetization methods, and groove depths of the workpiece, the groove depths on the surface of the workpiece and the leakage field generated by the workpiece will be “linear law” and “exponential law”, respectively [8,9,10]. The estimation method of the crack angle was discussed by I Uetake et al. [11]. When the lifting distance was small, the signal amplitude of flux leakage decreased with increasing crack width. At smaller crack angles, the ratio of peak-to-peak values (the ratio of the maximum value of magnetic flux to the minimum value) was larger. Furthermore, two methods for estimating the surface crack slope angle were proposed: one based on the leakage signal amplitude and the peak-to-peak ratio; the other on the gradient of the curve, the distance between the peak-to-peak ratio and the peak-to-valley value [12].

In the area of defect reconstruction and inversion for magnetic leakage detection, Zhenning Wu proposed an algorithm for defect reconstruction based on the iterative inversion method, designed an iterative strategy for the uniform sampling model, and realized the reconstruction of different defect sizes using this algorithm [13]. Wenhua Han et al. proposed a defect reconstruction model based on an improved artificial bee colony algorithm, which solves the radial basis function as a forward model for solving optimization problems in the inverse problem [14,15]. Fengzhu Ji formed the sample data through the simulation data obtained from experiments and 3D finite element simulation calculations, established the mapping relationship between the flux leakage signal of the defect and the 3D contour map of the defect, and realized the reconstruction of the 3D contour of the defect [16,17]. Ravan, M et al. proposed a new method for estimating the shape and depth profile of the opening of an arbitrary three-dimensional (3-D) defect in a magnetic leakage flux (MFL) measurement. The shape of the defect opening is estimated by using the Canny edge detection algorithm. An inversion procedure based on the spatial mapping (SM) method is then used in order to efficiently approximate the defect depth profile. Validation was also performed, and the results showed that the proposed methods all showed good agreement between the actual and estimated defect parameters [18,19]. J Chen proposed an iterative neural network for reconstructing three-dimensional defect distributions from triaxial MFL signals in pipe inspections. A radial basis function neural network is utilized as a forward model to predict the MFL signal for a given defect profile and update the defect profile based on a combination of gradient descent and simulated annealing during iterative inversion. The accuracy of the proposed inversion procedure was demonstrated in estimating the profiles of different defects in steel tubes [20].

Compared with the finite element method, the magnetic dipole method does not require the establishment of a complex mesh model and can directly analyze simple rectangular defects. The analytical expressions for the notch leakage signals of momentary, V-shaped, and combined notches were developed by Peng-Peng Shih and verified with the results available in the literature [21,22]. Dehui Wu studied the relationship between the leakage magnetic field distribution of defects and the magnetization angle, and solved the leakage magnetic signal in three dimensions [23,24,25]. Wenhua Han et al. designed a radial basis function neural network forward model based on magnetic dipole theory for irregular and complex three-dimensional leakage defects, and proposed an iterative algorithm based on the gravitational search algorithm, through which the defects can be reconstructed, which can effectively reduce the depth error and reconstructing time [26]. Mandache C and Clapham L quantitatively analyzed the relationship between the defect length and the leakage signal, developed a quantitative model, and experimentally verified it to achieve the inversion of the leakage signal [27,28]. D. Minkov and Y. Takeda, analyzed the magnetic charge distribution of irregular cross-sectional defects on the basis of the magnetic dipole model and concluded that the magnitude of the magnetic charge density was proportional to the depth of the defect [29].

In practical leak detection engineering applications, it is important to interpret the size of the defect by means of the leak signal. The mathematical relationship between defect size and the leakage signal provides a theoretical basis for the quantification of defects tracked by leak detection. Most of the current research into the forward solution of the leakage signal is based on the traditional magnetic charge model. The widely-used magnetic dipole model assumes that the magnetic charge is uniformly distributed on the sidewalls of the defect with a fixed constant density. However, in practical applications, the magnetic charge distribution is not entirely uniform, leading to discrepancies between the traditional calculation formula and actual detection results. These factors can affect the accuracy and effectiveness of magnetic dipole analysis. In this paper, on the basis of the traditional magnetic dipole model, considering the magnetic charge distribution that changes with the location of different depths, this replaces the previous formula for the magnetic charge density with a fixed value, and solves the rectangular defects with different geometric characteristics and builds an electromagnetic excitation detection platform for experimental comparison purposes.

## 2. Materials and Methods

### 2.1. Pipeline Magnetic Flux Leakage Detection Principle

**Figure 1:**
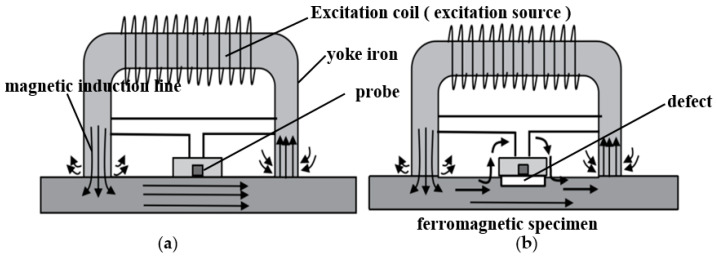
(**a**) is the schematic diagram of the magnetic line propagation path when there is no defect, and (**b**) is the case with defect.

Before starting magnetic flux leakage testing, the material to be tested must first be magnetized. The magnetic circuit comprises the excitation source, the yoke iron, and the ferromagnetic specimen to be inspected [30,31]. When there are no surface defects on the material, the magnetic induction lines inside the material uniformly pass through. However, if defects of varying sizes exist on the material’s surface, their magnetic permeability will change, resulting in a distortion of the magnetic induction line propagation path. This distortion causes some of the magnetic induction lines at the defect to leak outside the specimen, forming a leakage field. Different types of defects generate magnetic leakage signals with different amplitudes, polarities, and shapes. Figure 1a shows the tube wall without defects, and Figure 1b shows the tube wall when a defect exists. By further conditioning the detected magnetic flux leakage signal, the size of the defect can be determined.

### 2.2. Defect Analysis Modeling

A magnetic dipole of width a located between *x*_1_ and *x*_2_ in the XOY plane produces a magnetic potential at the point (*x*, *y*) in the two-dimensional plane from *x*_2_ to *x*_1_ as:

(1) $$\varphi =\int_{x_{1}}^{x_{2}}\left(-\frac{\sigma }{2\pi \mu_{0}}\right)\ln\sqrt{{\left(x-x_{0}\right)}^{2}+y_{0}{}^{2}}dx_{0}$$

where *μ*_0_ is the relative magnetic permeability of the magnetic dipole and σ is the magnetic charge density of the magnetic dipole.

Expansion of Equation (1) yields:

(2) $$\begin{array}{l} \varphi =(\frac{\sigma }{2\pi \mu_{0}})S(x,y)\\ S(x,y)=\left[\begin{array}{l} (x-x_{1})\ln\sqrt{{\left(x-x_{1}\right)}^{2}+y^{2}}\\ -(x-x_{2})\ln\sqrt{{\left(x-x_{2}\right)}^{2}+y^{2}}\\ +y\arctan(\frac{x-x_{1}}{y})\\ -y\arctan(\frac{x-x_{2}}{y})+(x-x_{1}) \end{array}\right] \end{array}$$

**Figure 2:**
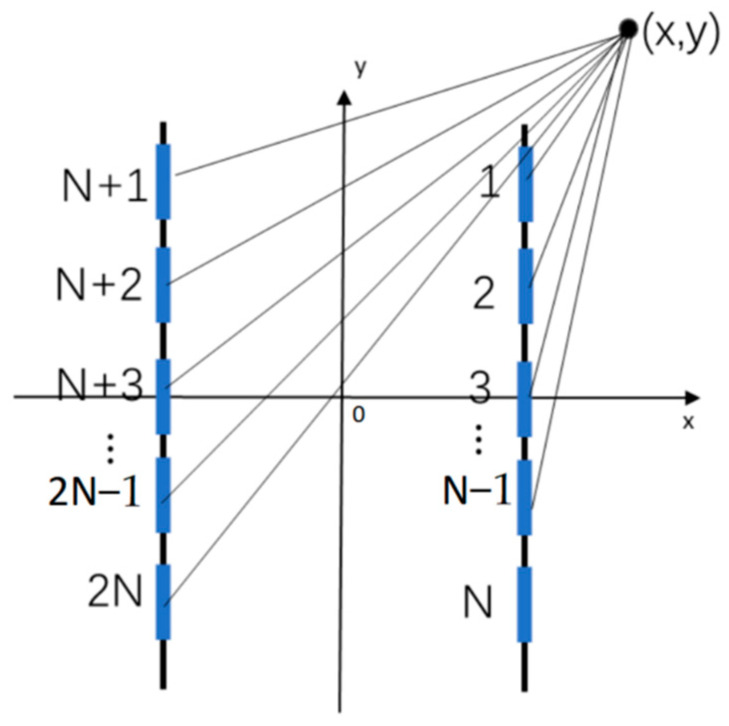
Schematic diagram of magnetic dipole line.

Let there exist two parallel magnetic dipoles with equal positive and negative magnetic charges in two-dimensional space, as shown in the Figure 2, there exists any point (*x*, *y*) in space, the magnitude of the magnetic potential generated by the magnetic dipole at that point can be regarded as the accumulation of several small segments of the magnetic dipole, the magnetic dipole is divided into 2N narrow strips, then, the magnitude of the magnetic potential at the point (*x*, *y*) can be expressed as.

(3) $$\varphi =\sum_{i=1}^{2N}\frac{\sigma (i)}{2\pi \mu_{0}}S(x-X_{i},y-Y_{i})$$

where *X*_i_, *Y*_i_ denotes the position of the center point of the *i*th narrow block long bar.

Let $S_{i,j}=S(x-X_{i},Y-Y_{i}^{})$, obtain a system of 2N linear equations, then the equipotential magnetic potential V on the two magnetic dipoles can be expressed as:

(4) $$\left[\begin{array}{cccc} S_{1,1} & S_{1,2} & \cdots & S_{1,2N}\\ S_{2,1} & S_{2,2} & \cdots & S_{2,2N}\\ \vdots & \vdots & \cdots & \vdots \\ S_{2N,1} & S_{2N,2} & \cdots & S_{2N,2N} \end{array}\right]\left[\begin{array}{c} \sigma_{1}\\ \sigma_{2}\\ \vdots \\ \sigma_{2N} \end{array}\right]=\left[\begin{array}{c} V_{1}\\ V_{2}\\ \vdots \\ V_{2N} \end{array}\right]$$

Since magnetic dipoles carry the same number of positive and negative magnetic charges with opposite magnetism, they can be equated as V_1_ = V_2_ = … V_N_ = 1, V_N+1_ = V_N+2_ = … V_2N_ = −1, then, the above equation can be transformed into:

(5) $$\left[\begin{array}{cccc} S_{1,1} & S_{1,2} & \cdots & S_{1,2N}\\ S_{2,1} & S_{2,2} & \cdots & S_{2,2N}\\ \vdots & \vdots & \cdots & \vdots \\ S_{2N,1} & S_{2N,2} & \cdots & S_{2N,2N} \end{array}\right]\left[\begin{array}{c} \sigma_{1}\\ \sigma_{2}\\ \vdots \\ \sigma_{2N} \end{array}\right]=\left[\begin{array}{c} 1\\ 1\\ \vdots \\ -1 \end{array}\right]$$

**Figure 3:**
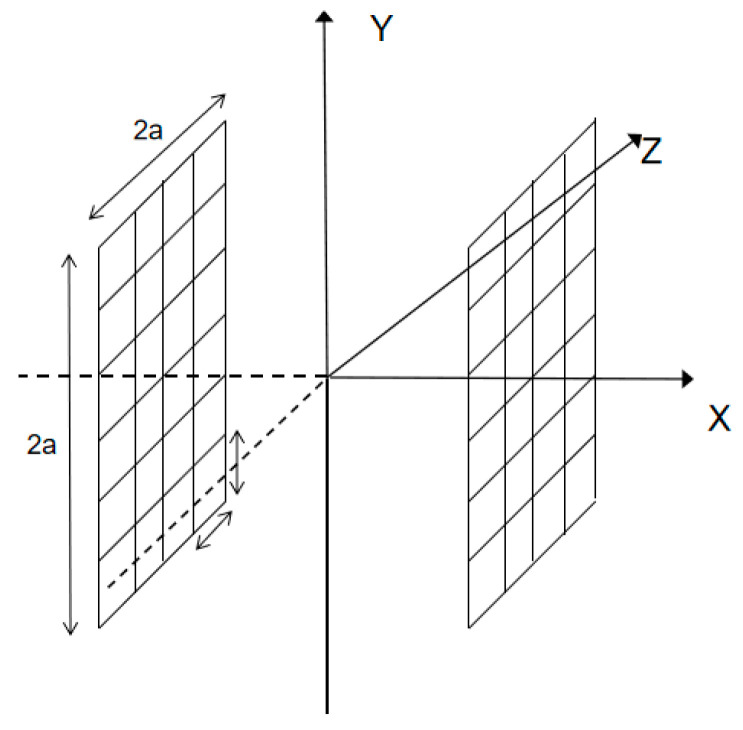
Schematic diagram of three-dimensional magnetic dipole surface.

Based on the realization principle of two-dimensional magnetic charge distribution, a three-dimensional model of the defect is established, the magnetic dipole line is expanded into a magnetic dipole plane, and the magnetic dipole plane is divided into squares of equal area. As shown in Figure 3, taking the center of each square as the relative position of the whole small square, let there exist two magnetic dipole surfaces with an equal number of positive and negative magnetic charges. The side length of the cross-section is divided into N parts on average, and it is divided into N^2^ small squares with equal blocks. The left and right sections are N^2^ blocks. Now, only one side of it is discussed, and each small square is numbered, and the magnetic charge density of each small square is expressed as $\sigma_{i}$. It is expressed by a matrix, if we use V_i,j_ to denote the magnetic potential at the center point of each small square, it is deduced from the formula that the magnetic potential at a point in space is:

(6) $$\begin{array}{l} \varphi =\int_{-a}^{a}dx\int_{-a}^{a}dy\frac{\sigma_{i}}{4\pi \mu_{0}}(\frac{1}{\sqrt{{\left(x-x_{0}\right)}^{2}+{\left(y-y_{0}\right)}^{2}+{\left(z-\frac{b}{2}\right)}^{2}}}-\frac{1}{\sqrt{{\left(x-x_{0}\right)}^{2}+{\left(y-y_{0}\right)}^{2}+{\left(z+\frac{b}{2}\right)}^{2}}})\\ =\sum_{\begin{array}{l} i=1\\ j=1 \end{array}}^{N}\frac{\sigma_{i}}{4\pi \mu_{0}}S(X_{i},Y_{j})\\ \left[\begin{array}{cccc} S_{1,1} & S_{1,2} & \cdots & S_{1,N}\\ S_{2,1} & S_{2,2} & \cdots & S_{2,N}\\ \vdots & \vdots & \cdots & \vdots \\ S_{N,1} & S_{N,2} & \cdots & S_{N,N} \end{array}\right]\left[\begin{array}{cccc} \sigma_{1,1} & \sigma_{1,2} & \cdots & \sigma_{1,N}\\ \sigma_{2,1} & \sigma_{2,2} & \cdots & \sigma_{2,N}\\ \vdots & \vdots & \cdots & \vdots \\ \sigma_{N,1} & \sigma_{N,2} & \cdots & \sigma_{N,N} \end{array}\right]=\left[\begin{array}{cccc} V_{1,1} & V_{1,2} & \cdots & V_{1,N}\\ V_{2,1} & V_{2,2} & \cdots & V_{2,N}\\ \vdots & \vdots & \cdots & \vdots \\ V_{N,1} & V_{N,2} & \cdots & V_{N,N} \end{array}\right] \end{array}$$

**Figure 4:**
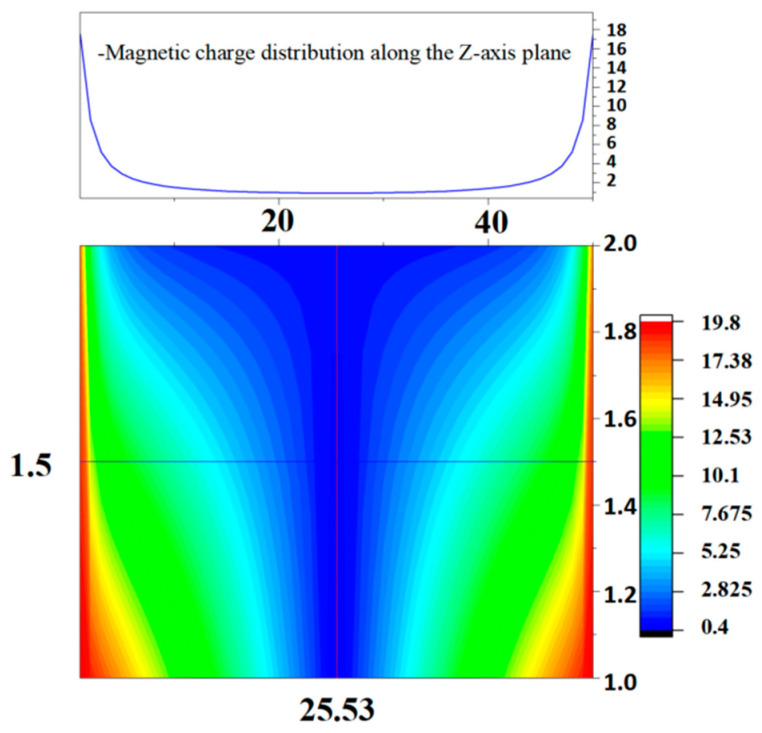
Schematic diagram of magnetic charge distribution.

Since the magnetic charges of any small square in the cross-section are equal and of opposite polarity, it can be regarded as an equipotential surface, and then all elements in V can be made to be 1, and thus the magnetic charge density of each small square in the density matrix can be found as a proportion of the whole surface. The solution is found to be more consistent with the edge effect as the number of cut copies increases. Setting N as 100, the center line along the *z*-axis on one of the cross-sections is selected, and the magnetic charge distribution along the line is analyzed as shown in the figure. As shown in Figure 4, the magnetic charge density shows a trend of having a high edge and low middle in the cross-section, which indicates that the actual distribution of magnetic charge is not completely uniformly distributed by the edge effect.

The traditional magnetic dipole model considers that the magnetic charge distribution presents a uniform distribution and its distribution formula is constant. Based on the distribution law found in the edge effect, the charge distribution model is improved on the basis of the traditional magnetic charge model, so that the equation of the magnetic charge density in this is:

(7) $$\sigma_{s}=\frac{2.65\mu_{0}(h/b+1)}{2\pi (h/(\mu_{r}z^{2}b)+1)}H_{0}$$

where $\mu_{0}$ is the relative magnetic permeability of the material, h is the defect depth, *b* is the defect width, *H*_0_ is the magnetization intensity, and *z* is the relative depth position from the edge.

**Figure 5:**
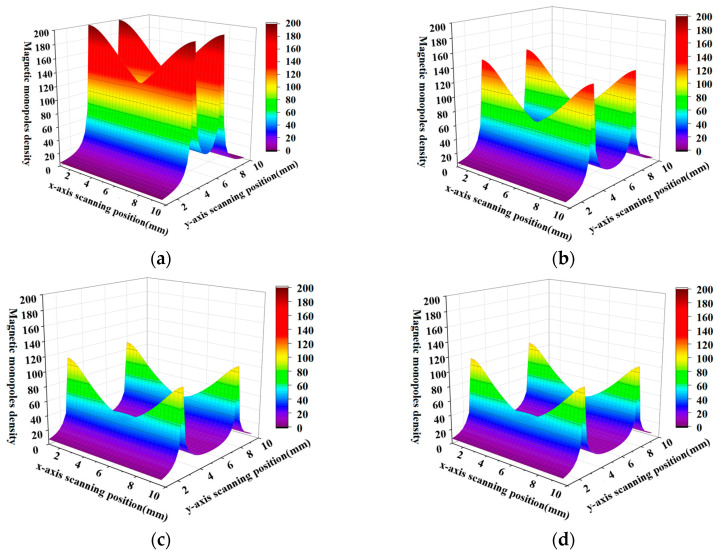
(**a**–**d**) show the trend of magnetic charge distribution on the plane when the plane spacing gradually increases, respectively.

When the spacing of the defect side wall is 5, 7, 9, 11, the magnetic charge distribution cloud diagram of the defect side wall is as follows:

When the X and Y axes are extended, the magnetic charge distribution exhibits a maximum at the edge, which is consistent with the magnetic charge distribution’s edge effect. The analysis of Figure 5 demonstrates that, according to this magnetic charge distribution model, the magnetic charge distribution peak is situated at the boundary, gradually declines with the increase in width, and presents a maximum at the edge.

In the ideal state, the magnetic charge is distributed on the side wall of the defect, which is composed of two magnetic charges with equal numbers and in different directions. According to the theory of magnetic dipoles, magnetic dipoles appear in pairs, and every point in the magnetic field is generated by a pair of magnetic dipoles with opposite polarity. When there is external magnetization, the magnetic dipole will rotate. According to the calculation formula of magnetic potential, the expression of the magnetic induction intensity of magnetic dipole can be obtained:

(8) $$B=\frac{\sigma }{4\pi }\frac{\vec{r}}{\mu_{0}r^{3}}$$

In Equation (8), *μ*_0_ is the vacuum permeability. *r* is the distance between magnetic dipoles, and σ is the magnetic charge density.

Set a rectangular defect of length 2*c*, width 2*b*, depth *h*, tube wall thickness *d* (unit/mm), and the magnetization field *H*_0_ direction parallel to the XOY plane, the magnetic field intensity generated at the point o(*x*, *y*, *z*) is:

(9) $$H=\frac{\sigma_{rs}}{2\pi \mu_{0}r^{2}}\vec{r}$$

According to the three-dimensional magnetic dipole model, the defective two-wall source point assumed (*x*_i_, *y*_i_, *z*_i_), the defective two-wall leakage magnetic field signal H formed at the spatial field point o(*x*, *y*, *z*), and the magnetic dipole model of the triaxial component is the binary integral of the defective wall magnetic charge, the magnetic dipole theoretical model obtains the following integral expression:

(10) $$\left\{\begin{array}{l} H_{x1}=\frac{2.65\mu_{0}(h/b+1)H_{0}}{4\pi^{2}(h/(\mu_{r}zb)+1)}\int_{-h}^{0}\int_{-c}^{c}\frac{x-x_{m}dy_{i}dz_{i}}{{\left[{\left(x-x_{i}\right)}^{2}+{\left(y-y_{i}\right)}^{2}+{\left(z-z_{i}\right)}^{2}\right]}^{\frac{3}{2}}}\\ H_{x2}=\frac{2.65\mu_{0}(h/b+1)H_{0}}{4\pi^{2}(h/(\mu_{r}zb)+1)}\int_{-h}^{0}\int_{-c}^{c}\frac{x+x_{m}dy_{i}dz_{i}}{{\left[{\left(x-x_{i}\right)}^{2}+{\left(y-y_{i}\right)}^{2}+{\left(z-z_{i}\right)}^{2}\right]}^{\frac{3}{2}}}\\ H_{y1}=\frac{2.65\mu_{0}(h/b+1)H_{0}}{4\pi^{2}(h/(\mu_{r}zb)+1)}\int_{-h}^{0}\int_{-c}^{c}\frac{y-y_{m}dy_{i}dz_{i}}{{\left[{\left(x-x_{i}\right)}^{2}+{\left(y-y_{i}\right)}^{2}+{\left(z-z_{i}\right)}^{2}\right]}^{\frac{3}{2}}}\\ H_{y2}=\frac{2.65\mu_{0}(h/b+1)H_{0}}{4\pi^{2}(h/(\mu_{r}zb)+1)}\int_{-h}^{0}\int_{-c}^{c}\frac{y+y_{m}dy_{i}dz_{i}}{{\left[{\left(x-x_{i}\right)}^{2}+{\left(y-y_{i}\right)}^{2}+{\left(z-z_{i}\right)}^{2}\right]}^{\frac{3}{2}}}\\ H_{z1}=\frac{2.65\mu_{0}(h/b+1)H_{0}}{4\pi^{2}(h/(\mu_{r}zb)+1)}\int_{-h}^{0}\int_{-c}^{c}\frac{z-z_{m}dy_{i}dz_{i}}{{\left[{\left(x-x_{i}\right)}^{2}+{\left(y-y_{i}\right)}^{2}+{\left(z-z_{i}\right)}^{2}\right]}^{\frac{3}{2}}}\\ H_{z2}=\frac{2.65\mu_{0}(h/b+1)H_{0}}{4\pi^{2}(h/(\mu_{r}zb)+1)}\int_{-h}^{0}\int_{-c}^{c}\frac{z+z_{m}dy_{i}dz_{i}}{{\left[{\left(x-x_{i}\right)}^{2}+{\left(y-y_{i}\right)}^{2}+{\left(z-z_{i}\right)}^{2}\right]}^{\frac{3}{2}}} \end{array}\right.$$

The leakage fields of the different directional components of the two sidewalls perpendicular to the magnetization direction are separately solved, and the final complete defect leakage field magnitude is obtained after accumulation.

(11) $$\left\{\begin{array}{l} H_{x}=H_{x2}-H_{x1}\\ H_{y}=H_{y2}-H_{y1}\\ H_{z}=H_{z2}-H_{z1} \end{array}\right.$$

On the basis of this model, the corresponding dimensional and parametric information is input, the size of the lift-off value is set to 1 mm, and the magnetic permeability parameter is selected as *μ* of 3000; the above parameters are brought into the solved model, and the *x* and *y* directional components are obtained as shown below. The radial and axial components are consistent with the conventional leakage signal, which illustrates the feasibility of the model.

### 2.3. Calculation Results Analysis Comparison

**Figure 6:**
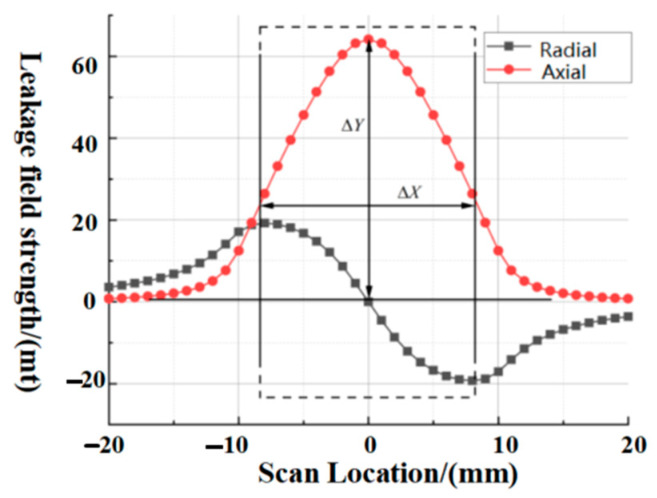
The results of the improved magnetic charge model calculation, where the black line shows the radial leakage component and the red line shows the axial leakage component.

**Figure 7:**
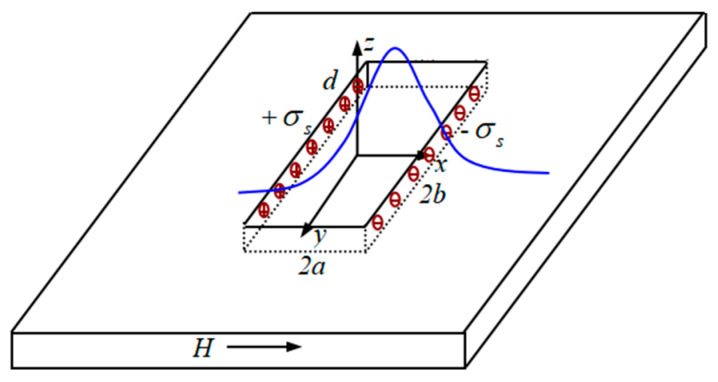
Schematic of the leakage field generated under the improved magnetic charge model when defects are present.

According to the result of Equation (11), the relevant parameters are brought in and a numerical calculation program is written to design the current rectangular defect with a width of 10 mm and a depth of 1.6 mm. The software calculation environment is a Windows system and the numerical analysis is calculated by Matlab software. The relative permeability of the pipe wall is 3000, and the applied magnetization field strength H is set to 3000 A/m, as shown in Figure 6 and Figure 7. The magnetization direction is the same as the axial direction of the defect.

**Figure 8:**
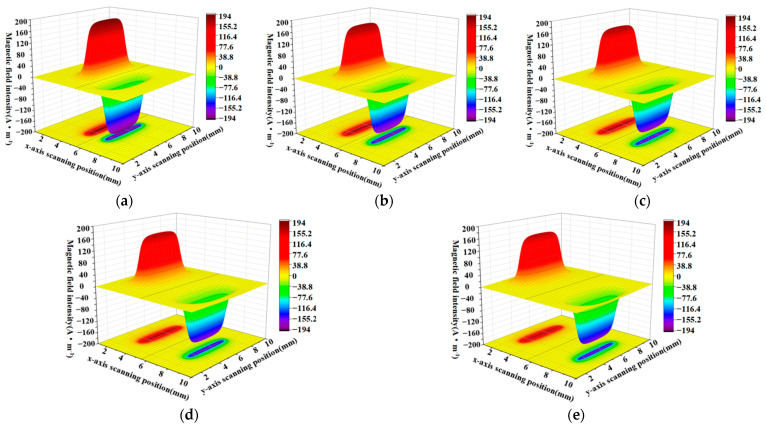
The radial distribution characteristics of the leakage magnetism in 3D space when the length of the defect is changed. (**a**–**e**) corresponding to the energy distribution of the three-dimensional radial leakage signal for defects of 4 mm, 6 mm, 8 mm, 10 mm and 12 mm in length, in that order.

**Figure 9:**
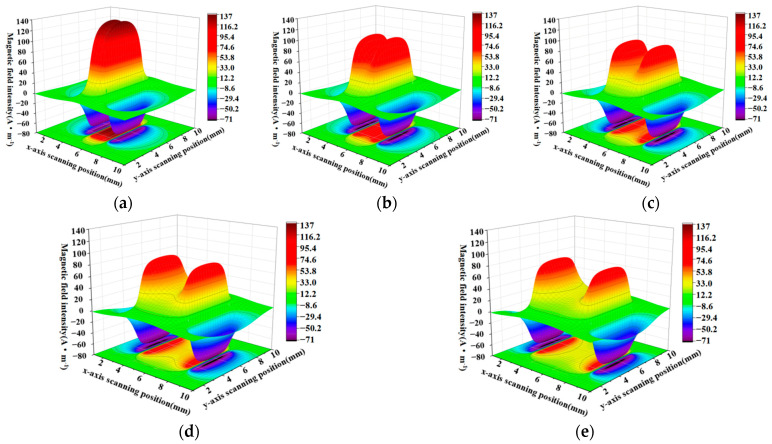
The characteristics of the axial distribution of the leakage magnetism in three dimensions when the length of the defect is changed. (**a**–**e**) corresponding to the energy distribution of the three-dimensional axial leakage signal for defects of 4 mm, 6 mm, 8 mm, 10 mm and 12 mm in length, in that order.

When the defect lengths are 4 mm, 6 mm, 8 mm, 10 mm, and 12 mm, respectively, the calculated radial components and axial leakage fields of the three-dimensional distribution of rectangular defects under the improved non-uniform magnetic charge model are shown in Figure 8 and Figure 9.

**Figure 10:**
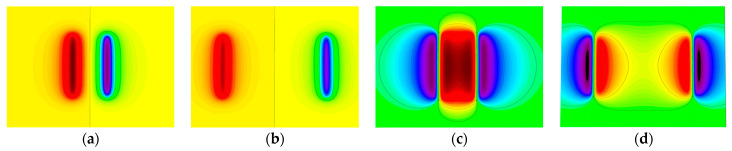
The projected images of the 3D leakage field on the ground for the maximum and minimum lengths. (**a**,**b**) show the projection images of the radial leakage component, and (**c**,**d**) show the projection images of the axial leakage component.

Figure 8a–e shows the variation of the spatial radial leakage signal of the defect as the length of the defect increases; it can be seen that the distance between the peaks and valleys of the axial leakage signal gradually increases as the length of the defect increases. The magnitude of the crest and trough also decreases with increasing length. Figure 9a–e shows the axial signal of the spatial leakage of defects at different lengths, which follows a similar pattern to the radial leakage signal. The ground projections of the axial and radial leakage signals at the maximum and minimum defect lengths are also selected in Figure 8 and Figure 9. As shown in Figure 10, Figure 10a shows the projection of the radial leakage signal at the minimum defect length, Figure 10b shows the projection of the radial leakage signal at the maximum defect length, Figure 10c shows the projection of the axial leakage signal at the minimum defect length, and Figure 10d shows the projection of the axial leakage signal at the maximum defect length. 

**Figure 11:**
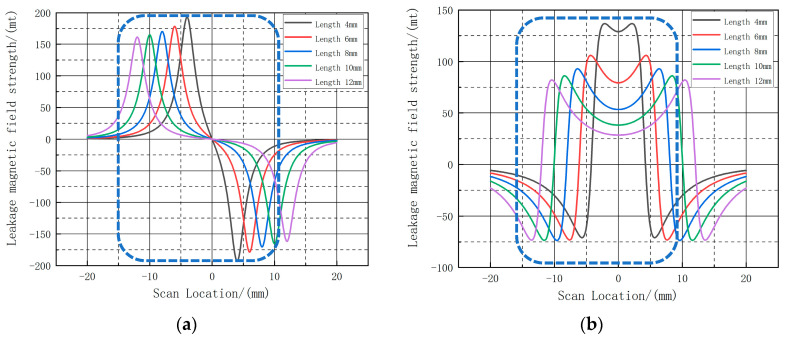
Variation of the leakage magnetic field distribution at the center intercept line for defects with different lengths. (**a**) shows the radial leakage component, and (**b**) shows the axial leakage component. The peaks and troughs of the radial and axial components are shown in the blue text boxes.

In order to accurately analyze the relationship between the leakage signal and the length of the defect, the calculated leakage signal at the center of the defect is selected and the curves of the axial and radial leakage signals after selection are shown in Figure 11:

As shown in Figure 11, the different color curves represent the leakage signal at the center of the defect at different lengths. As shown in Table 1, the amplitude of the radial leakage signal in Figure 11 varies from 162.67 A·m^−1^ to 193.14 A·m^−1^ and the amplitude of the axial leakage signal varies from 82.09 A·m^−1^ to 136.78 A·m^−1^. Similarly, as the length of the defect increases, the peak-to-valley spacing of the radial leakage signal gradually increases and the amplitude decreases. The axial leakage signal varies in the same way as the radial leakage signal. The change in length only affects the magnitude of the curve and the spacing between peaks and troughs, not the shape of the curve.

**Figure 12:**
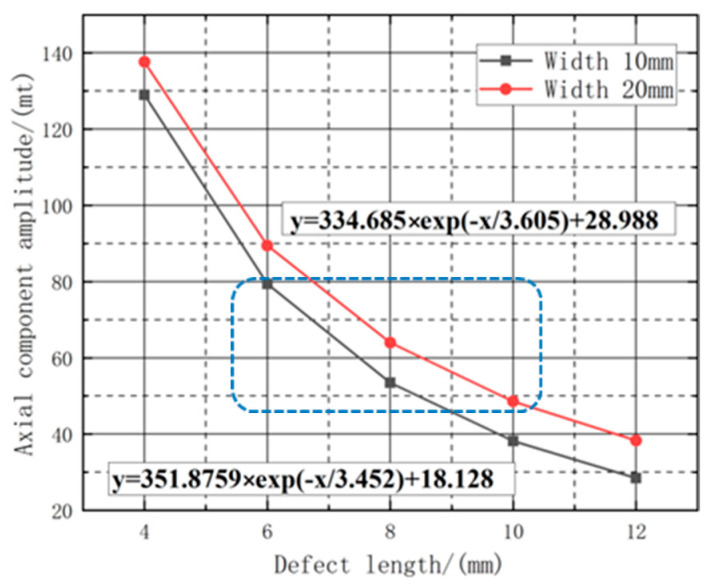
Fitting curves of different lengths with axial component leakage field amplitude.

Further analysis was conducted on the dimensional characteristics of the defects, as well as the influence of different defect lengths on the leakage signal. In order to extract the defect widths of 10 mm and 20 mm, a range of length changes between 4 mm and 12 mm were selected. The axial peak of the detection signal was measured, and a first-order exponential function (f = A × (1 − e^(−b × X))) was employed to fit the mathematical relationship between the peak and the defect length. In this function, A represents the amplitude of signal attenuation, while b indicates the rate of signal attenuation. The attenuation characteristics of the leakage signal are shown in Figure 12. 

As illustrated in Figure 12, the leakage signal exhibits an exponential decay pattern with increasing length. By utilizing the fitted curves, the attenuation amplitude of the detected signal can be calculated for various lifting values. Table 2 presents the attenuation amplitude of the axial leakage signal for different widths and lengths.

**Figure 13:**
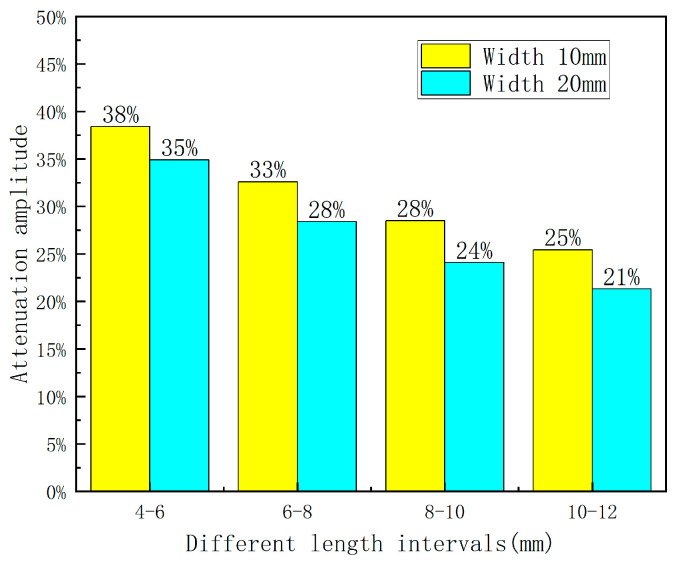
Attenuation amplitude diagram.

Based on Figure 13, the decay rate of the axial leakage signal reaches its peak and gradually decreases as the defect length increases within different length variations. Additionally, wider defect widths result in lower decay rates within the same length variation interval, implying that the width can partially suppress amplitude variations of the leakage signal. Furthermore, defected areas with larger widths exhibit higher sensitivity to amplitude variations associated with the length of the leakage signal.

**Figure 14:**
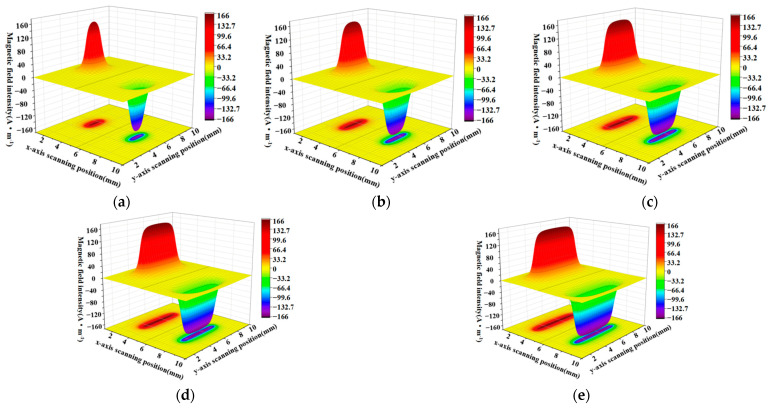
Three-dimensional defect leakage magnetic distribution of the radial leakage component as the width increases. (**a**–**e**) correspond to the three-dimensional spatial distribution of the radial leakage field for widths of 4 mm, 6 mm, 8 mm, 10 mm and 12 mm, in that order.

**Figure 15:**
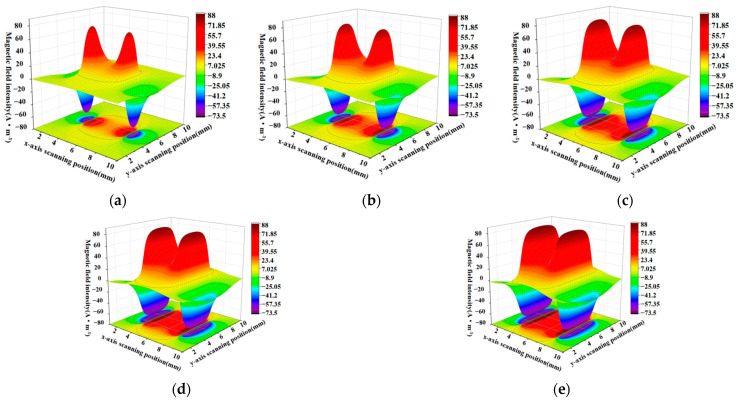
(**a**–**e**) correspond to the three-dimensional spatial distribution of the axial leakage field for widths of 4 mm, 6 mm, 8 mm, 10 mm and 12 mm, in that order.

Similarly, when the defect length is fixed at 10 mm and the width of the defect is 4 mm, 6 mm, 8 mm, 10 mm, and 12 mm, the calculation results of the rectangular defect under the improved non-uniform magnetic charge distribution model are shown in Figure 14 and Figure 15.

**Figure 16:**
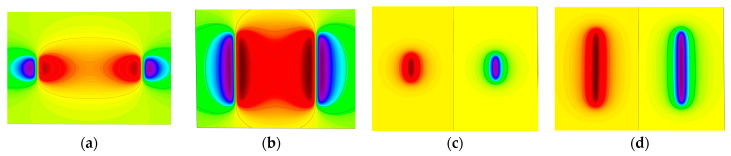
Projected images of the 3D leakage field on the ground for the maximum and minimum widths. (**a**,**b**) show the projection images of the radial leakage component, and (**c**,**d**) show the projection images of the axial leakage component.

From a comparison of Figure 14a–e and Figure 15a–e, it can be seen that the increase in width will cause the cross-sectional area of the three-dimensional magnetic flux leakage field to increase along the longitudinal direction, and the peak and trough of the axial magnetic flux leakage signal and the radial magnetic flux leakage signal also gradually increase. As shown in Figure 16, by selecting the smallest width and the largest width, regarding the ground projection, it can be seen whether it is the axial component or the radial component, the proportion of the peak area significantly increases with the increase of the width.

**Figure 17:**
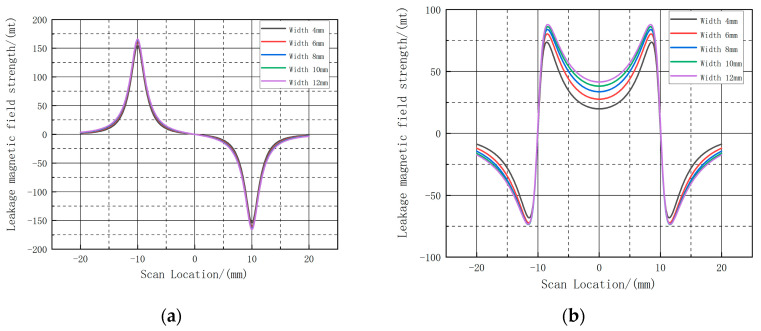
Variation of the leakage magnetic field distribution at the center intercept line for defects with different widths. (**a**) shows the radial leakage component, and (**b**) shows the axial leakage component.

At this time, the same selection of the center of the defect out of the three-dimensional intercept, to obtain each group of defects at the intercept of the axial component and radial component, as shown in Figure 17, with the increase in the width of the defect, the defect of the radial leakage component amplitude change is small, showing a slight increase, the axial leakage component amplitude increase is more obvious, while the shape of the leakage curve does not change; as shown in Table 3, the axial peak fluctuation range: 19.97 A·m^−1^~41.6027 A·m^−1^; radial peak and valley fluctuation range: 152.829 A·m^−1^~164.193 A·m^−1^. The fluctuation range of the axial peak is 19.97 A·m^−1^~41.6027A·m^−1^; the fluctuation range of the radial peak and valley is 152.829 A·m^−1^~164.193A·m^−1^.

**Figure 18:**
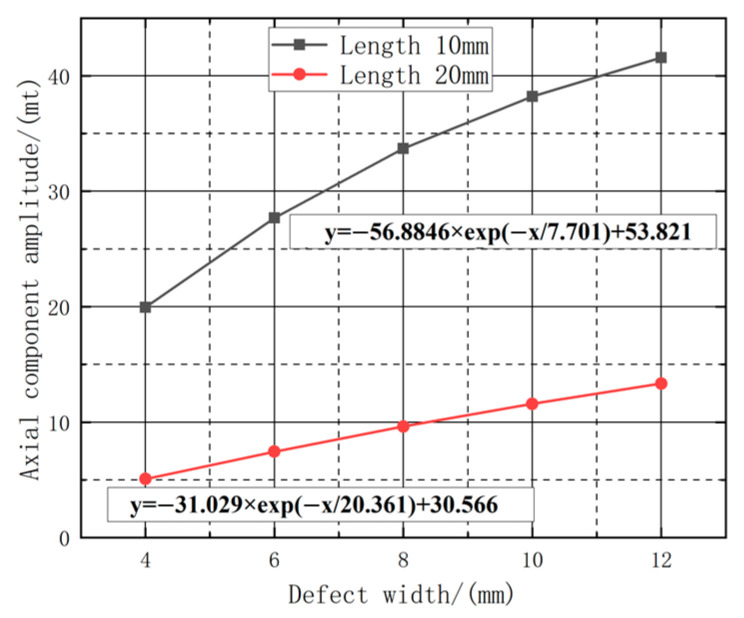
Fitting curves of different widths with axial component leakage field amplitude.

In order to investigate the law of the effect of the defect width on the change in amplitude under different defect lengths, the relationship between the change of defect width and the amplitude of the axial magnetic leakage component under the fixed defect lengths of 10 mm and 20 mm, respectively, the fitted image is shown in Figure 18. The amplitude of the magnetic leakage signal exponentially increases with the increase in width. Using the fitted curve to calculate the increased amplitude of the detection signal under different widths, the increased amplitude of the axial leakage signal under different lengths and width variations is tabulated as shown in Table 4.

**Figure 19:**
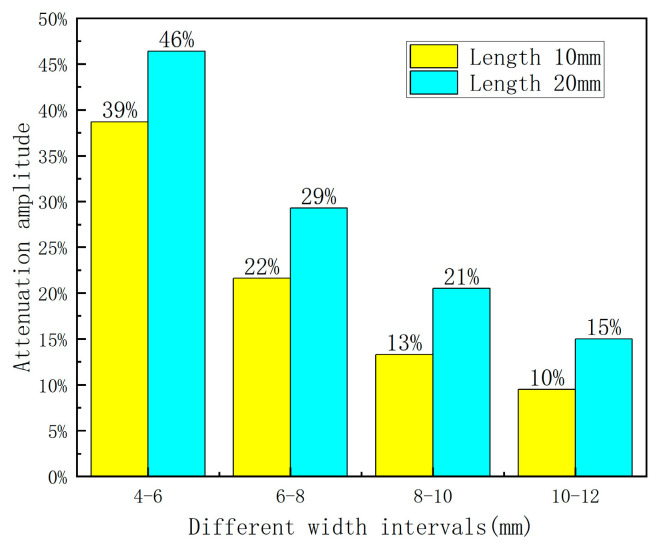
Rate of increase of width and defect amplitude at different lengths.

Based on the data presented in Table 4, it can be determined that on the premise of fixed length and depth, as the width variation intervals change, the peak increase rate of the axial magnetic flux leakage signal declines with increasing defect length. Moreover, As shown in Figure 19, the amplitude value also decreases as the defect length increases. Additionally, within the same width variation interval, the increase rate becomes faster as the defect length increases, indicating that length somehow influences the amplitude change of the magnetic flux leakage signal. In essence, longer defects are characterized by a more sensitive amplitude change in the magnetic flux leakage signal with respect to width correlation.

**Figure 20:**
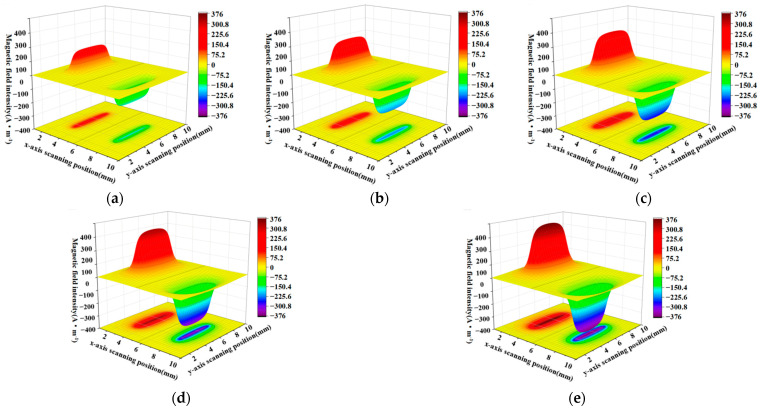
Three-dimensional defect leakage magnetic distribution of the radial leakage component as the depth increases. (**a**–**e**) show the variation of the radial 3D leakage field when the depths are 1.6 mm, 2.4 mm, 3.2 mm, 4 mm and 4.8 mm respectively.

**Figure 21:**
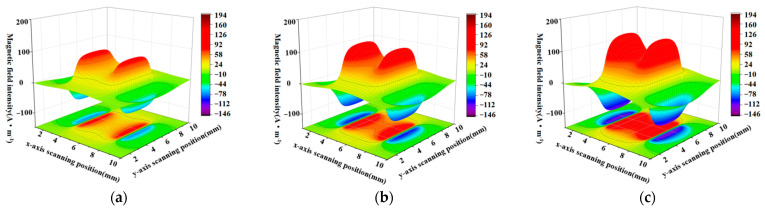

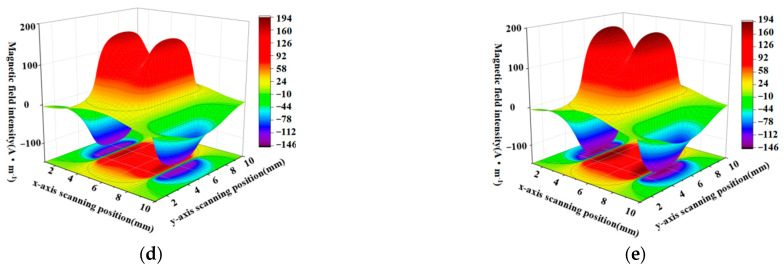
Three-dimensional defect leakage magnetic distribution of the axial leakage component as the depth increases. (**a**–**e**) show the variation of the axial 3D leakage field when the depths are 1.6 mm, 2.4 mm, 3.2 mm, 4 mm and 4.8 mm respectively.

Analyzing the effect of defect depth on the leakage magnetic signal, when the defect length is fixed at 10 mm and the width of the defect is fixed at 10 mm, the calculated results of the three-dimensional leakage magnetic field of rectangular defects under the improved non-uniform magnetic charge distribution for defect depths of 1.6 mm, 2.4 mm, 3.2 mm, 4 mm, and 4.8 mm are shown in Figure 20 and Figure 21. From Figure 20a–e, compared with Figure 21a–e, it is evident that an increase in depth results in a noteworthy rise in the peak of the defect leakage magnetic field in the axial and radial directions. Moreover, the projected areas of the red and blue regions on the ground also increase, and the intensities of the colors darken, indicating that the extreme values are becoming more pronounced.

**Figure 22:**
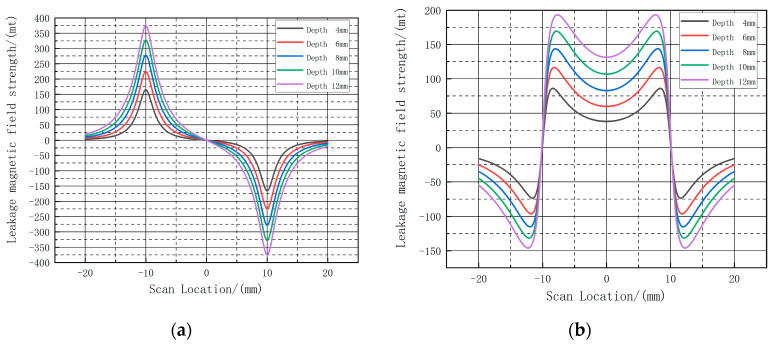
Variation of the leakage magnetic field distribution at the center intercept line for defects with different depths. (**a**) shows the radial leakage component, and (**b**) shows the axial leakage component.

At this point, the same selection of the center of the defect out of the three-dimensional intercept line is used to obtain each group of defects at the intercept line of the axial component and radial component, as shown in Figure 22.

**Figure 23:**
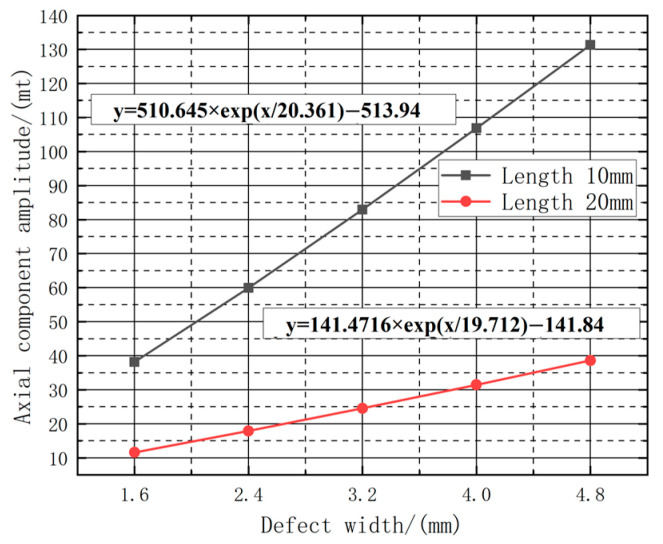
Fitting curves of different depths with axial component leakage field amplitude.

As shown in Table 5, with the length and width of the defect fixed, the increase in the depth of the defect will cause an increase in the magnitude of the axial leakage signal and the radial leakage signal; the magnitude of the axial leakage signal fluctuates from 38.239 A·m^−1^ to 131.394 A·m^−1^, and the magnitude of the radial leakage signal fluctuates from 163.541 A·m^−1^ to 372.177 A·m^−1^, respectively. The length of the fixed defect is 10 mm, 20 mm, the width is fixed at 10 mm. Regarding the relationship between the variation of the depth of the defect and the amplitude of the axial leakage component, the fitted image is shown in Figure 22; the amplitude of the leakage signal exponentially increases with the increase of the depth. The fitted curve is used to calculate the amplitude of the increase in the detection signal at different depths. As shown in Figure 23, the depth of the defect and the magnitude of the axial signal shows an exponentially increasing relationship. The fitted curve is used to calculate the increase in the detection signal under different lengths; to obtain different lengths, the depth of the axial leakage signal increase is shown in the amplitude table, as shown in Table 6.

**Figure 24:**
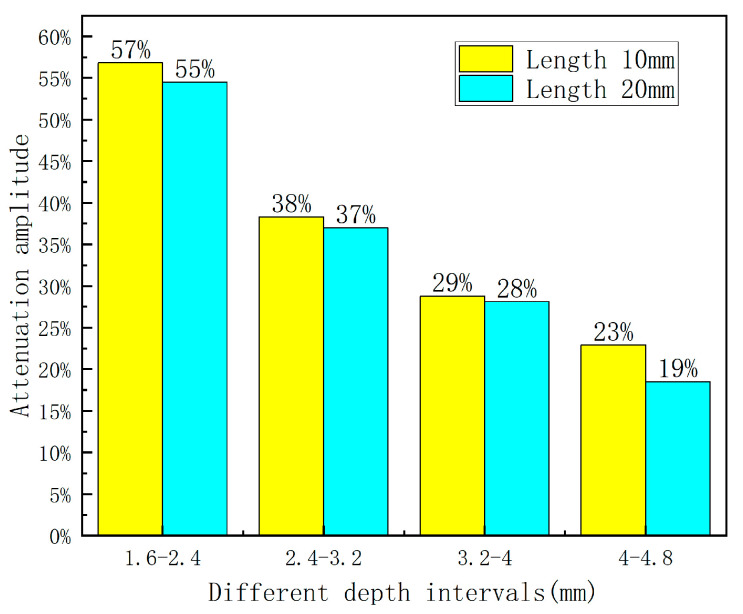
Diagram of the increase.

As per Table 6, the analysis of variation in the axial leakage signal at different lengths reveals that the peak increase rate decreases with defect depth and that the value magnitude slightly reduces with defect length. As shown in Figure 24, within the same length variation interval, shorter defect lengths exhibit faster increase rates, indicating a minimal suppressive effect of length on the amplitude change in the leakage signal. Additionally, shorter defect lengths demonstrate greater sensitivity to amplitude changes associated with width.

## 3. Results and Discussion

In order to verify the degree of agreement between the analytical model and the actual leakage magnetic field of the defect obtained from the measured leakage magnetic signal, as well as to assess the characteristics of the magnetic signal, a leakage magnetic detection experimental platform was built.

**Figure 25:**
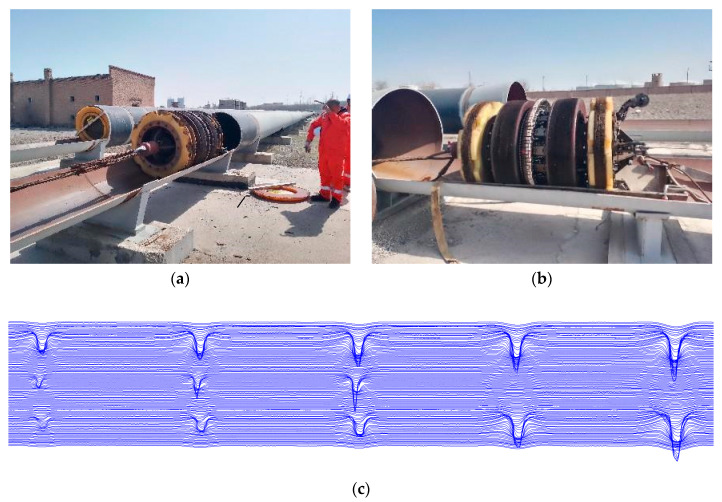
(**a**) shows the schematic diagram of the experimental platform, (**b**) shows the schematic diagram of the defective specimen, and (**c**) shows the axial signal of the detected defect leakage.

**Figure 26:**
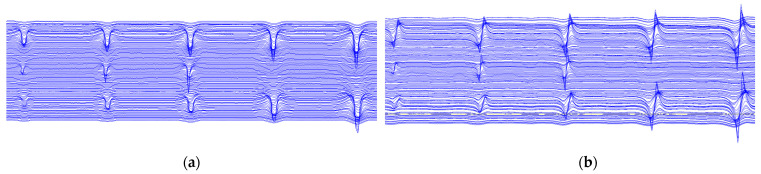
(**a**) shows the radial leakage component, and (**b**) shows the axial leakage component.

The experiments were carried out using a 1219 in-pipe detector; the experimental platform is shown in Figure 25. The experiments were carried out by means of permanent magnet excitation, a skin bowl, a pipe with defects, a mileage wheel, a computer, and other components of the internal leakage detection experimental platform. The propulsion speed was controlled at 1.5 m/s. The specific experimental site is shown in Figure 25:

The advancing of the detector is controlled by forming a propulsive force through the skin bowl, isolating the gas in front of and behind the detector, acquiring its leakage signal through a fixed placed Hall sensor, conditioning the signal through a data acquisition box, digital to analog conversion, transferring the data to the host computer, and converting it into a curve through analysis software and displaying it on the computer. The defect is grooved and the cross-section is shown in Figure 25c. The signal characteristics after each excitation are shown in Figure 26, which are similar to the model calculations, with the axial leakage signal showing a curve with one trough and one peak.

After building, the experimental platform can be measured on the defect injury and the measurement results from Figure 26 can be seen; the measurements are averaged over three replicate experiments and the numerical results are within 10% error between each tow. The defect signal has a pair of radial wave peaks and valleys distributed in the two tips of the defect, the axial component in a certain length range appears at a great value located in the center of the defect, and is the same as the theoretical calculation. In terms of a comparison of the magnitude of the magnitude after the solution and the actual measurement, the comparison results are shown in Table 7 and Table 8.

**Figure 27:**
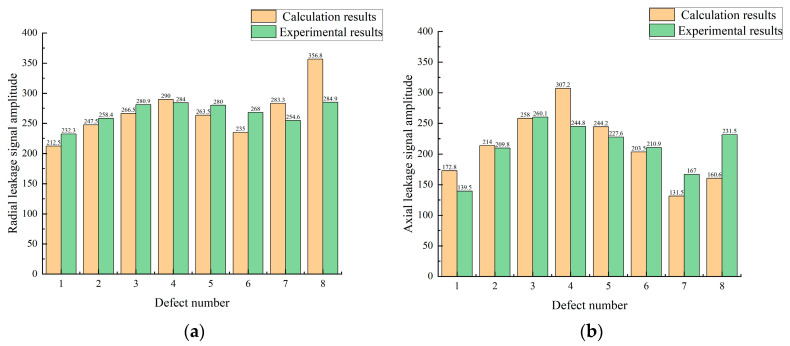
(**a**) shows a comparison between the experimental data and the calculated radial signal amplitude, while (**b**) shows a comparison of the axial signal amplitude.

The magnitude of the experimentally obtained leakage signal was compared with the results obtained from the model calculations, as shown in the Figure 27. After analysis, it was found that the average error for the radial leakage signal was 9.34% and the average error for the axial leakage signal was 14.3%. The model was better adapted for small-sized defects and the error was enhanced for large-sized defects.

Then, in order to verify the accuracy under the improved non-uniformly distributed magnetic charge model, the results of the calculations under the non-uniform magnetic charge model were compared with those under the conventional uniform magnetic charge model (which considers the magnetic charge distribution to be a constant value related to the size of the defect). The environmental correction parameters relevant to the experiment were entered into the program and the results were calculated by forward resolution. The results are shown in the figure.

**Figure 28:**
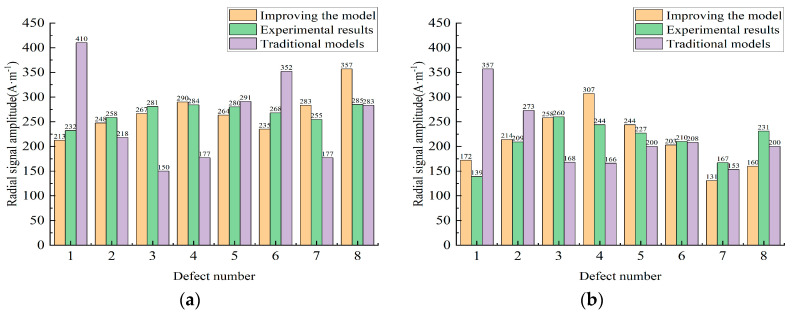
(**a**,**b**) show a comparison of the conventional uniform magnetic charge model and the improved non-uniform magnetic charge with experimental results.

As shown in Figure 28, the improved forward-solving model improved and increased the accuracy of the solution for specific size defects to some extent compared to the conventional magnetic charge model. For large defects, the improved model provided a more significant improvement in computational accuracy, with less error compared to the traditional model.

**Figure 29:**
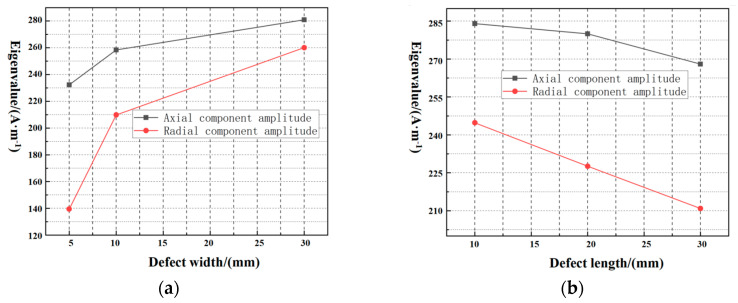
(**a**) shows the fitting curve of width dimension and axial component amplitude, and (**b**) shows the fitting curve of length dimension and axial component amplitude.

After fitting the measured results for defects with different length and width variations, it was found that an increase in length causes a decrease in eigenvalues and an increase in width causes an increase in eigenvalues, as shown in Figure 29. The series of laws obtained from the three-dimensional forward solver model based on rectangular defects are consistent with the experimental detection results and with engineering experience, which verifies the correctness of the proposed model to describe the leakage magnetic field distribution of defects in the pipe wall. The validity of this magnetic dipole 3D modeling can thus be illustrated.

## 4. Conclusions

In this study, based on the traditional uniform magnetic charge distribution model, an improved analytical model of rectangular defects is established, and the non-uniformly distributed magnetic charge model, in which the magnetic charge distribution changes with position, is used to replace the traditional uniform magnetic charge distribution by the three-dimensional magnetic dipole theory. The leakage signal distribution of rectangular defects of different sizes is solved and analyzed, and the following conclusions are obtained.

- The length variation of the defect is correlated with the peak and valley values of the axial leakage components. The increase in the long axis of the defect causes an increase in the spacing of the peak and valley values and a decrease in the amplitude of the axial and radial leakage components.
- The increase in defect width causes an increase in the amplitude of the axial and radial components. Both show a rising exponential relationship under the same conditions; the smaller the length of the defect by this effect is more obvious, thus the greater the increase.
- The increase in the depth of the defect causes an increase in the amplitude of the axial and radial components. The two show a rising exponential relationship; the larger the width of the defect under the same conditions, the more sensitive to this effect and the greater the increase.
- The results were compared with the leakage magnetic field signals of rectangular defects of the same geometry under actual experiments, and the solved leakage magnetic signals agreed with the variation pattern obtained from the measured experimental results. The forward-solving model provides relevant parameters for the quantification of defects and the evaluation of the remaining life of the pipe. The analytical model provides a theoretical basis for the reconstruction and quantification of defects in practical engineering applications, and provides a reference basis for the prediction and evaluation of defects.

## Figures and Tables

**Table 1:** Variation of leakage signal characteristics at different lengths.

Defect Length (mm)	4	6	8	10	12
ΔX	4	6	8	10	12
ΔY	5.6	7.7	9.5	11.7	13.7
Radial component peak	136.78	105.95	92.96	86.16	82.09
Axial component peak	193.14	178.67	170.46	165.25	162.67

**Table 2:** Table of amplitude decay rates at different lengths.

Length Variation Interval	Width 10 mm	Width 20 mm
4–6 mm	38.4%	34.9%
6–8 mm	32.6%	28.4%
8–10 mm	28.5%	24.1%
10–12 mm	25.44%	21.33%

**Table 3:** Variation of leakage signal characteristics at different widths.

Defect Length (mm)	4	6	8	10	12
ΔX	10	10	10	10	10
ΔY	11.5	11.4	11.7	11.7	11.6
Radial component peak	73.63	80.3	83.83	86.16	87.84
Axial component peak	154.52	161.39	164.02	165.25	165.91

**Table 4:** Table of amplitude increase rates at different widths.

Length Variation Interval	Width 10 mm	Width 20 mm
4–6 mm	38.7%	46.4%
6–8 mm	21.6%	29.3%
8–10 mm	13.3%	20.5%
10–12 mm	9.5%	15%

**Table 5:** Variation of leakage signal characteristics at different depths.

Defect Length (mm)	1.6	2.4	3.2	4	4.8
ΔX	10	10	10	10	10
ΔY	11.5	11.9	11.8	11.9	12
Radial component peak	86.16	116.45	143.66	169.05	193.33
Axial component peak	165.25	224.54	227.97	327.68	374.76

**Table 6:** Table of amplitude increase rates at different depths.

Length Variation Interval	Length 10 mm	Length 20 mm
1.6–2.4 mm	56.8%	54.5%
2.4–3.2 mm	38.3%	37%
3.2–4 mm	28.8%	28.1%
4–4.8 mm	22.9%	18.5%

**Table 7:** Comparison of amplitude of axial leakage components.

Defect Size	Calculation Results	Experimental Results
1. 20 mm × 5 mm × 10 mm	212.5	232.3
2. 20 mm × 10 mm × 10 mm	247.5	258.4
3. 20 mm × 30 mm × 10 mm	266.5	280.9
4. 10 mm × 20 mm × 10 mm	290	284
5. 20 mm × 20 mm × 10 mm	263.5	280
6. 30 mm × 20 mm × 10 mm	235	268
7. 40 mm × 40 mm × 4.4 mm	283.3	254.6
8. 40 mm × 40 mm × 6.6 mm	356.8	284.9

**Table 8:** Radial leakage component amplitude comparison.

Defect Size	Calculation Results	Experimental Results
1. 20 mm × 5 mm × 10 mm	172.8	139.5
2. 20 mm × 10 mm × 10 mm	214	209.8
3. 20 mm × 30 mm × 10 mm	258	260.1
4. 10 mm × 20 mm × 10 mm	307.2	244.8
5. 20 mm × 20 mm × 10 mm	244.2	227.6
6. 30 mm × 20 mm × 10 mm	203.5	210.9
7. 40 mm × 40 mm × 4.4 mm	131.5	167
8. 40 mm × 40 mm × 6.6 mm	160.6	231.5

## Data Availability

For ethical and privacy reasons, research data are not publicly available; please contact the corresponding author if required.
